# Early Detection Screening of Cognitive Decline in Patients Over 60 Years: ELDERCARE Study

**DOI:** 10.3233/JAD-231295

**Published:** 2024-03-05

**Authors:** Salvatore Putignano, Luigi Forgione, Mariano Fusco, Attilio Giacummo, Elisa Magli, Saverio Marino, Raffaele Marzano, Daria Putignano, Francesco Santamaria, Micaela Spatarella, Vincenzo Santagada

**Affiliations:** a Associazione Geriatri Extraospedalieri a favore di Anziani Svantaggiati (A.G.E.A.S.), Naples, Italy; b Ordine dei Farmacisti della Provincia di Napoli, Naples, Italy

**Keywords:** Alzheimer’s disease, cognitive dysfunction, elderly, frail elderly, observational study

## Abstract

**Background:**

Dementia is the fourth leading cause of death in people >  65 years old in western countries.

**Objective:**

This cross-sectional assisted survey aimed to evaluate a multidisciplinary team approach of specialists of the Associazione Geriatri Extraospedalieri a favore di Anziani Svantaggiati and pharmacists to facilitate progress in the early identification and management of cognitive decline in patients >  60 years.

**Methods:**

A multidisciplinary team conducted this cross-sectional assisted survey. Patients (>60 years) with independent and/or assisted walking, subjective memory impairment, mild cognitive impairment or mild Alzheimer’s disease (AD) who regularly attended pharmacies underwent the survey. An internal medical examination, a cardiovascular visit, and a short neuropsychological evaluation were conducted for each patient. Demographic, anamnestic, and clinical data were collected anonymously.

**Results:**

279 eligible patients underwent the screening phase. 44% were overweight, 23% obese and 29% hypertensive. 62% of cases showed alterations of supra-aortic trunk with different percentages of stenosis. The neuropsychological evaluation highlighted that 67% of cases were normal according to age and education level, while 18% were in a state condition of cognitive frailty. Mild/moderate cognitive decline, or probably AD, was identified in 14% of cases.

**Conclusions:**

A multidisciplinary collaboration between pharmacists and specialist medical doctors is essential in early identification of prodromal symptoms of cognitive impairment and AD. The Prompt detection of the condition in this group of patients allowed the specialists to recommend in-depth diagnostic tests and follow-up procedures to slow the course of the disease. This would give time to carry out adequate caregiver training.

## INTRODUCTION

Dementia is the fourth leading cause of death in people over the age of 65 years in western countries, accounting for 55 million cases worldwide (0.8% of the general population) and is expected to reach 150 million cases in 2050. In industrialized countries, the prevalence of dementia exceeds 8% in the over 60-year-old population and 20% in the over 80-year-old population. The incidence is 10 million new cases/year, costing approximately US$1 trillion/year [1–3].

Alzheimer’s disease (AD) and, to a lesser extent, vascular dementia are the leading causes of age-related cognitive decline. AD and vascular dementia may have common risk factors (e.g., hypertension, diabetes, smoking), and vascular damage contributes to the aggravation of the clinical manifestations of AD [4]. AD accounts for 60–70% of cases [1–3].

In Italy, over 1 million people are affected by dementia, 600,000 of whom have AD [5].

Frailty is strongly associated with dementia risk. Kelaiditi et al. defined cognitive frailty as the simultaneous presence of physical frailty and mild cognitive impairment (Clinical Dementia Rating = 0.5) in the absence of dementia or pre-existing brain disorders [6]. The International Academy on Nutrition and Ageing and the International Association of Gerontology and Geriatrics have defined cognitive impairment as a “state of reduced cognitive reserve that is different from physiological brain ageing and is characterised by potential reversibility” [7].

The absence of resolutive therapies for dementias and the expected further population aging draw worrying epidemiological projections. Furthermore, cardiovascular disease is the leading cause of death, and the vascular risk profile changes with age from predominantly cardiovascular to predominantly cerebrovascular [8].

Therefore, greater attention to prevention is required by correcting modifiable risk factors and detecting pathological cognitive decline symptoms early.

In this context, the Eldercare study was designed in collaboration between the Association of Non-Hospital Geriatrics (AGEAS) and the Council of Pharmacists of Naples to identify cognitive decline early in a population over the age of 60 years.

AGEAS is a non-profit organization that provides the opportunity to make visits with medical specialists and fosters a culture of prevention that encourages a change in lifestyles for a healthy life.

AGEAS carries out continued specific clinical screening and educational initiatives. The Eldercare study is part of this activity by providing free medical consultations, visits, and diagnoses for people in different pharmacies of the Campania region during ad hoc events. The pharmacy is the place of first and immediate response to health needs, a point of information dissemination and screening initiatives, allowing the recruitment of many people in a short time.

The collaboration between scientific societies, such as AGEAS and the Council of Pharmacists, is strategic and bases its strength on the central role of territorial reference of the pharmacy.

## MATERIALS AND METHODS

### Study design and aims

This is a cross-sectional assisted interview survey designed by geriatricians and conducted on a sample population over 60 years of age with the following aims:

– Test a multidisciplinary team on cognitive decline management.
– Early identify the condition of cognitive decline (cognitive frailty).

### Methods

**Fig. 1:**
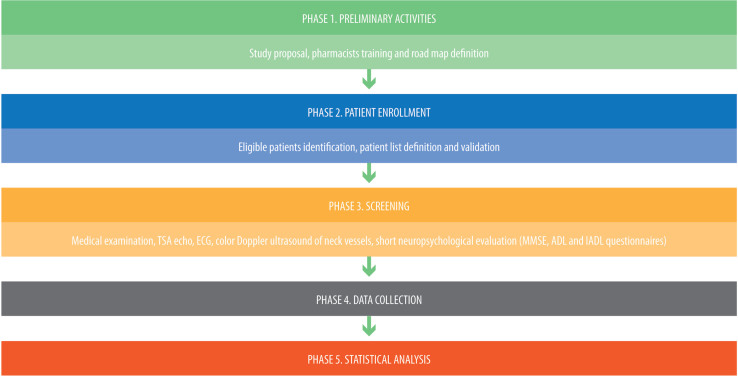
Project phases.

The survey is part of a project that consists of five phases (Fig. 1).

#### Phase 1. Preliminary activities

Multidisciplinary team set up.

A multidisciplinary team consisting of AGEAS specialists and pharmacists of the Province of Naples was set up to design the study, define the tools and plan the activities.

Study proposal and pharmacist training.

The survey was presented to pharmacists during an ad hoc meeting, and adhesions were collected. Later, preparatory meetings were held to train pharmacists on the criteria for patient recruitment and to manage the patient selection form.

Road map.

The team defined the road map, identifying the study settings and data collection dates.

#### Phase 2. Patient enrolment

AGEAS organized prevention-related events, and the pharmacists enrolled the patients.

The pharmacists preidentified the patients two weeks before the start of the screening phase according to the following criteria:

Eligibility criteria.

Subjects who regularly go to local pharmacies and agreed to participate in the screening.

Inclusion criteria.

• Age >  60 years
• Independent and/or assisted walking
• Subjective memory impairment
• Signature of informed consent (written informed consent was obtained from all patients per the Helsinki Declaration for human studies).

Exclusion criteria.

• Age <  60 years
• Dementia diagnosis

#### Phase 3. Screening

A camper to carry out the screening visits and equipped with all the necessary equipment was set up by the Council of Pharmacists of Naples. The survey was carried out by AGEAS specialists in campervans of the Council of Pharmacists in dedicated areas close to the participating pharmacies. Each patient underwent the following:

• Medical examination of weight, height, SpO_2_%, blood pressure;
• Supra-aortic trunk echo-Doppler measuring intimal thickness and presence of plaques;
• Electrocardiogram (ECG) to evaluate rhythm and ischemic anomalies;
• Color Doppler ultrasound of neck vessels;
• Short neuropsychological evaluation through administering the Mini Mental State Examination (MMSE) [9], activities of daily living (ADL) [10] and the Lawton Instrumental Activities of Daily Living Scale (IADL) [11] questionnaires.

Based on the scores obtained from the administration of the MMSE questionnaire, the cognitive profile of the patients was defined as:

– Normal according to age and education level (score between 24 and 30 points)
– Cognitive frailty (score higher than 24 points but physical frailty)
– Mild cognitive impairment (score between 19 and 23 points)
– Moderate (score between 10 and 18 points)

#### Phase 4. Data collection

Demographic, anamnestic, and clinical data were collected anonymously during screening in ad hoc designed Case Report Forms (CRFs).

#### Phase 5. Statistical analysis

Patient characteristics are described using frequencies and percentages for categorical variables or medians and interquartile ranges in the case of continuous variables. Comparisons between groups are purely descriptive. Data processing was performed using the Statistical Package for the Social Sciences (SPSS) Statistics Standard.

## RESULTS

The study was carried out in 17 pharmacies in the Province of Naples.

**Fig. 2:**
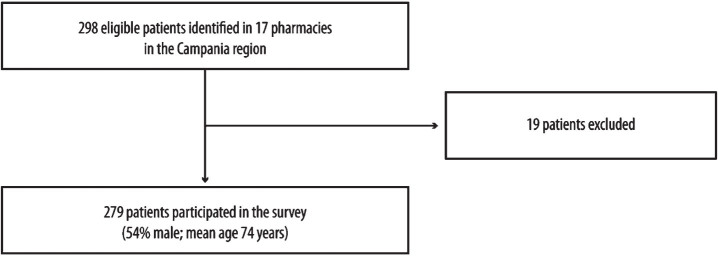
Patient enrolment.

298 patients were eligible to participate in the survey. Of these, 19 subjects <  60 years were identified and, according to the exclusion criteria, were not considered in the data analysis (Fig. 2). The analysis was conducted on 279 subjects, 53,8% male, with a mean age of 74 years (range: 60–89; Table 1).

**Table 1:** Sample characteristics

Characteristics	*n* = 279, *n* (% )
Male	150 (53.8)
Age (y), mean±SD	74±6
Age groups
60–70	91 (32.6)
70–80	147 (52.7)
>80	41 (14.7)
BMI (kg/m^2^)
<25	86 (30.8)
25–30	123 (44.1)
≥30	64 (22.9)
Blood pressure (mmHg)
<120/80	199 (71.3)
>120/80	80 (28.7)

The medical examination revealed that 44.1% of the patients were overweight, 22.9% were obese, and 28.7% were hypertensive (Table 1).

ECG was normal or had rhythm disturbances not worthy of further investigation in 94% of patients. Supra-aortic trunk echo-Doppler examination showed alterations in 62.3% of cases with different percentages of stenosis (69.4% with lesions <  35% ; 30.1% with lesions between 35% and 70% ; only one case with lesions >  70% ) (Table 2). Thyroid disorder was also identified, including enlarged thyroid lobes in 16% of cases and thyroid nodularity in 13% (data not shown).

**Table 2:** Clinical outcomes

Clinical outcomes	*n* (% )
Carotid lesions	173 (62.3)
<35%	120 (69.4)
35–70	52 (30.1)
≥70%	1 (0.6)
Cognitive profile
Normal according to age and education level	187 (67.0)
Cognitive frailty	50 (17.9)
Mild cognitive impairment	31 (11.1)
Moderate	8 (2.9)

A short neuropsychological evaluation highlighted that 67% of cases were normal according to age and education level, while 17.9% were in a condition of cognitive frailty. Mild and moderate cognitive decline was identified in 14% of cases (Table 2).

The most frequent risk factors were hypertension, polyarthrosis, dyslipidemia, epiaortic chronic obstructive arterial disease and diabetes, with a higher prevalence in patients with worse cognitive profiles (Table 3).

Cerebral vasculopathy was underestimated due to the subjective minimization of vague symptoms and lack of diagnostic investigation with brain imaging tests.

Polyarthrosis represented one of the most frequent risk factors, demonstrating that physical inactivity plays an important role in the development of cognitive decline.

The presence of two or more risk factors was more frequent with the deterioration of the cognitive profile. The most frequent combinations were hypertension–polyarthrosis and hypertension–dyslipidemia (25.1% and 23.5% in subjects with a normal cognitive profile, 50% and 37.5% in those with moderate cognitive decline, respectively) (Table 3).

The presence of risk factors and the patient’s clinical conditions have led clinicians to suggest new therapeutic and preventive strategies (i.e., supra-aortic trunk echo-Doppler) to reduce the cardiocerebrovascular risk as much as possible.

**Table 3:** Risk factors stratified by cognitive profile

Risk factor	Normal according to age		Cognitive		Mild cognitive		Moderate	
	and education level		frailty		impairment			
	*n*	%	*n*	%	*n*	%	*n*	%
Hypertension	106	56.7	37	74.0	22	71.0	7	87.5
Polyarthrosis	87	46.5	27	54.0	21	67.7	5	62.5
Dyslipidemia	68	36.4	21	42.0	17	54.8	4	50.0
Epiaortic chronic obstructive arterial disease	68	36.4	21	42.0	13	41.9	–	–
Diabetes	35	18.7	7	14.0	10	32.3	2	25.0
Ischemic heart disease	15	8.0	8	16.0	4	12.9	1	12.5
Chronic renal failure	7	3.7	3	6.0	2	6.5	–	–
COPD	7	3.7	3	6.0	–	–	–	–
Atrial fibrillation	4	2.1	1	2.0	3	9.7	1	12.5
Hyperuricemia	1	0.5	1	2.0	1	3.2	–	–
Cerebral vascular disease	1	0.5	–	–	–	<0.1	–	–
Hypertension–polyarthrosis	47	25.1	21	42.0	15	48.4	4	50.0
Hypertension–dyslipidemia	44	23.5	14	28.0	15	48.4	3	37.5
Hypertension–supra-aortic trunks	42	22.5	16	32.0	10	32.3	–	–
Diabetes–supra-aortic trunks	16	8.6	3	6.0	3	9.7	–	–
Diabetes–dyslipidemia	12	6.4	2	4.0	9	29.0	–	–
Hypertension–ischemic heart disease	8	4.3	8	16.0	3	9.7	1	12.5

## DISCUSSION

New healthcare models are required today due to demographic changes, i.e., the aging of the population and the impoverishment of the socioeconomic context for elderly patients suffering from chronic pathologies, increasing exponentially in all industrialized countries. According to the WHO, treatment for the elderly impacts more than 80% of healthcare costs [1, 12]. Therefore, a widespread network of professionals is essential to respond to these people’s needs [13].

With this cross-sectional assisted interview survey, the first at a national level, we wanted to test the value and the strength of multidisciplinary collaboration between pharmacists and specialists in managing elderly patients. The local pharmacy has been an excellent reference point to reach a large population. Pharmacists assist patients with chronic health conditions, and community pharmacies are an ideal setting for healthcare delivery. International evidence supports the key role of community pharmacy services for consumers with asthma, diabetes and cardiovascular conditions, opioid substitution, and preventative health (e.g., weight management, smoking cessation) [14]. Professional pharmacist services result in positive patient health outcomes. Indeed, health systems increasingly rely on community pharmacists to provide services in addition to traditional dispensing services. Finally, community pharmacy teams continued to provide essential services during the COVID-19 pandemic, playing a fundamental role in COVID-19 and influenza vaccinations [15].

In this context, the local pharmacy administered the survey free of charge to frail elderly patients for cognitive screening. In the current healthcare conditions, such screening would require multiple specialist visits and much time spent on this. Therefore, during this survey, a service tailored to elderly patients was provided, respecting socio-health needs and requirements.

In addition to this, specialists assessed cardio-cerebrovascular risk factors to identify patients with cognitive deficits early [16]. 62% of enrolled patients had carotid lesions. Then, our results suggest that supra-aortic trunk echo-Doppler should be included in the geriatric evaluation to allow the identification of cardiovascular alterations and the stratification of cardio-cerebrovascular risk. Moreover, patients with cardiovascular risk factors should undergo neuropsychological evaluation to allow adequate stratification of cardiovascular risk and cognitive decline [13].

### Conclusion

This study suggests the importance of multidisciplinary collaboration between pharmacists and specialist medical doctors to identify clinical conditions early and to allow the specialist to recommend in-depth diagnostic tests and follow-up procedures aimed at slowing down the course of the disease, especially in neurodegenerative disorders.

## AUTHOR CONTRIBUTIONS

Salvatore Putignano (Conceptualization; Data curation; Formal analysis; Writing – original draft; Writing – review & editing); Luigi Forgione (Writing – review & editing); Mariano Fusco (Writing – review & editing); Attilio Giacummo (Writing – review & editing); Elisa Magli (Writing – review & editing); Saverio Marino (Writing – review & editing); Raffaele Marzano (Writing – review & editing); Daria Putignano (Data curation; Formal analysis; Investigation; Writing – original draft; Writing – review & editing); Francesco Santamaria (Writing – review & editing); Micaela Spatarella (Writing – review & editing); Vincenzo Santagada (Conceptualization; Writing – original draft; Writing – review & editing).

## Data Availability

The data supporting the findings of this study are available within the article.

## References

[1] WHO (2015) The epidemiology and impact of dementia: Current state and future trends. Geneva:World Health Organization; Document WHO/MSD/MER/15.3, available at http://www.who.int/mental_health/neurology/dementia/dementiathematicbrief_epidemiology.pdf. Accessed March 8, 2017.

[2] Long S , Benoist C , Weidner W (2023) World Alzheimer Report 2023: Reducing dementia risk: Never too early, never too late. Alzheimer’s Disease International, London, England. https://www.alzint.org/u/World-Alzheimer-Report-2023.pdf. Accessed October 25, 2023.

[3] Alzheimer’s Disease International and WHO (2012) Dementia: A public health priority. Geneva: World Health Organization, http://www.who.int/mental_health/publications/dementia_report_2012/en/. Accessed March 8, 2017.

[4] Iadecola C , Duering M , Hachinski V , Joutel A , Pendlebury ST , Schneider JA , Dichgans M (2019) Vascular Cognitive Impairment and Dementia: JACC Scientific Expert Panel, J Am Coll Cardiol 73, 3326–3344. 31248555 10.1016/j.jacc.2019.04.034 PMC6719789

[5] Claudia P , Volpe U (2019) ‘Italy’, Dementia Care: International Perspectives. In Dementia Care: International Perspectives, Burns A, Robert P, eds. Oxford Academic, Oxford, UK. https://doi.org/10.1093/med/9780198796046.003.0025. Accessed February 10, 2023.

[6] Kelaiditi E , Cesari M , Canevelli M , van Kan GA , Ousset PJ , Gillette-Guyonnet S , Ritz P , Duveau F , Soto ME , Provencher V , Nourhashemi F , Salvà A , Robert P , Andrieu S , Rolland Y , Touchon J , Fitten JL , Vellas B IANA/IAGG(2013) Cognitive frailty: Rational and definition from an (I.A.N.A./I.A.G.G.) international consensus group. J Nutr Health Aging 17, 726–734. 24154642 10.1007/s12603-013-0367-2 PMC12878905

[7] Facal D , Maseda A , Pereiro AX , Gandoy-Crego M , Lorenzo-López L , Yanguas J , Millán-Calenti JC (2019) Cognitive frailty: A conceptual systematic review and an operational proposal for future research. Maturitas 121, 48–56. 30704565 10.1016/j.maturitas.2018.12.006

[8] Olvera Lopez E , Ballard BD , Jan A (2023) Cardiovascular disease. In StatPearls [Internet]. StatPearls Publishing, Treasure Island, FL. 30571040

[9] Monroe T , Carter M (2012) Using the Folstein Mini Mental State Exam (MMSE) to explore methodological issues in cognitive aging research. Eur J Ageing 9, 265–274. 28804426 10.1007/s10433-012-0234-8 PMC5547414

[10] Brorsson B , Asberg KH (1984) Katz index of independence in ADL. Reliability and validity in short-term care. Scand J Rehabil Med 16, 125–132. 6494836

[11] Graf C (2008) The Lawton instrumental activities of daily living scale, Am J Nurs 108, 52–62. quiz 62-63. 10.1097/01.NAJ.0000314810.46029.74 18367931

[12] Baruth JM , Gentry MT , Rummans TA , Miller DM , Burton MC (2020) Polypharmacy in older adults: The role of the multidisciplinary team. Hosp Pract (1995) 48, 56–62. 31900000 10.1080/21548331.2019.1706995

[13] Choi JY , Lee JY , Shin J , Kim CO , Kim KJ , Hwang IG , Lee YG , Koh SJ , Hong S , Yoon SJ , Kang MG , Kim JW , Kim JH , Kim KI (2022) COMPrehensive geriatric AsseSSment and multidisciplinary team intervention for hospitalised older adults (COMPASS): A protocol of pragmatic trials within a cohort, BMJ Open 12, e060913. 10.1136/bmjopen-2022-060913 PMC9345040 35914913

[14] Piquer-Martinez C , Urionagüena A , Benrimoj SI , Calvo B , Martinez-Martinez F , Fernandez-Llimos F , Garcia-Cardenas V , Gastelurrutia MA (2022) Integration of community pharmacy in primary health care: The challenge. Res Social Adm Pharm 18, 3444–3447. 35016847 10.1016/j.sapharm.2021.12.005

[15] Maidment I , Young E , MacPhee M , Booth A , Zaman H , Breen J , Hilton A , Kelly T , Wong G (2021) Rapid realist review of the role of community pharmacy in the public health response to COVID-19, BMJ Open 11, e050043. 10.1136/bmjopen-2021-050043 PMC8210681 34135054

[16] Fillit H , Nash DT , Rundek T , Zuckerman A (2008) Cardiovascular risk factors and dementia. Am J Geriatr Pharmacother 6, 100–118. 18675769 10.1016/j.amjopharm.2008.06.004

